# Application Potential of Lion’s Mane Mushroom in Soy-Based Meat Analogues by High Moisture Extrusion: Physicochemical, Structural and Flavor Characteristics

**DOI:** 10.3390/foods14193402

**Published:** 2025-10-01

**Authors:** Yang Gao, Song Yan, Kaixin Chen, Qing Chen, Bo Li, Jialei Li

**Affiliations:** Food Processing Research Institute, Heilongjiang Academy of Agricultural Sciences, Harbin 150086, China; gao20031026@163.com (Y.G.); 1261456355@163.com (S.Y.); 4228041@163.com (K.C.); chenqingchen163@163.com (Q.C.); blnky@163.com (B.L.)

**Keywords:** soy protein isolate, Lion’s mane mushroom, high-moisture extrusion, physicochemical characteristics, protein conformation, volatile flavor compounds, sensory property

## Abstract

The aim of this work was to systematically evaluate the effects of Lion’s Mane Mushroom powder (LMM, 0–40%) on the physicochemical properties, structural characteristics, and flavor profile of soy protein isolate-based high-moisture meat analogues (HMMAs). Optimal incorporation of 20% LMM significantly enhanced product quality by acting as a secondary phase that inhibited lateral protein aggregation while promoting longitudinal alignment, achieving a peak fibrous degree of 1.54 with dense, ordered fibers confirmed by scanning electron microscopy. Rheological analysis showed that LMM improved viscoelasticity (G′ > G″) through β-glucan; however, excessive addition (≥30%) compromised structural integrity due to insoluble dietary fiber disrupting protein network continuity, concurrently reducing thermal stability as denaturation enthalpy (_Δ_H) decreased from 1176.6 to 776.3 J/g. Flavor analysis identified 285 volatile compounds in HMMAs with 20% LMM, including 98 novel compounds, and 101 flavor metabolites were upregulated. The mushroom-characteristic compound 1-octen-3-ol exhibited a marked increase in its Relative Odor Activity Value of 18.04, intensifying mushroom notes. Furthermore, LMM polysaccharides promoted the Maillard reaction, increasing the browning index from 48.77 to 82.07, while β-glucan induced a transition in protein secondary structure from random coil to β-sheet configurations via intramolecular hydrogen bonding. In conclusion, 20% LMM incorporation synergistically improved texture, fibrous structure, and flavor complexity—particularly enhancing mushroom aroma. This research offers valuable insights and a foundation for future research for developing high-quality fungal protein-based meat analogues

## 1. Introduction

Sustained global population growth will lead to a substantial increase in total protein demand [1]. However, traditional livestock production, which supplies animal protein, faces significant limitations, including concerns over resource scarcity, environmental pollution, and animal welfare issues associated with its production processes [2]. From a human health perspective, chronic high consumption of red and processed meats has been implicated in the development of well-documented health issues, including obesity, type 2 diabetes, cardiovascular disease, stroke, and colorectal cancer [3]. Bruinsma predicts that by 2050, animal product production will double, from 229 billion kg for a population of 6 billion in 2000 to 465 billion kg for a population of 9.1 billion [4]. Nevertheless, reducing meat demand is highly challenging due to its fundamental role in human diets, necessitating the development of sustainable meat alternatives [4]. In this context, the combined challenges posed by population growth and dietary transitions have accelerated the emergence of the meat analogue market. Currently, a critical research priority and development focus is the utilization of plant proteins and novel sustainable protein sources to create nutritionally balanced meat analogue products that accurately replicate the quality attributes of animal meat—including structure, texture, and flavor—thereby meeting market demand for high-quality alternatives.

Among various meat analogue processing technologies, high-moisture extrusion (HME) has established itself as the leading technology due to its low energy consumption, simple process, scalability, good stability, and high fibrousness [5]. The core difference between high-moisture extrusion technology and low-moisture extrusion technology is the moisture content of the material and the final product form during processing. Low-moisture extrusion (usually less than 40% moisture) is performed at high temperatures and pressures to produce a dry, tight “semi-finished” tissue protein that must be rehydrated before use. High-moisture extrusion (usually 40–80% moisture content) can directly create a “finished product” with a moist, fibrous texture that is very similar to the muscle fibers of real meat in a single extrusion process [5]. Soy protein, as a significant plant protein resource, has become the preferred material for manufacturing meat analogues and is widely utilized in HME processes. This preference is largely attributed to its superior gelation properties, rich nutritional profile, as well as relatively low cost, particularly in purified forms such as soy protein concentrate (SPC) and soy protein isolate (SPI) [6,7]. Edible mushrooms comprise a rich profile of essential nutrients, such as protein, lipids, carbohydrates, minerals, and vitamins. Notably, mushroom protein exhibits high nutritional value and beneficial bioactivity [8,9]. Furthermore, mushrooms are rich in sulfur-containing amino acids (e.g., methionine and cysteine), which are key precursors to compounds that impart desirable meat-like characteristics [10]. Consequently, mushroom protein has emerged as a promising alternative protein source, recognized for its high quality, ease of production, and cost-effectiveness. Current research suggests incorporating edible mushrooms as raw materials for meat analogues. For instance, Yuan et al. prepared meat analogues using SPI combined with shiitake (*Lentinula edodes*), oyster (*Pleurotus ostreatus*), and ink cap (*Coprinus comatus*) mushrooms, or an equal mixture thereof, via extrusion puffing, subsequently using these as ingredients in sausage development [11]. Mohamad et al. investigated the physicochemical properties of extrudates by adding oyster mushrooms (*Pleurotus ostreatus*) to meat analogues based on soy protein fabricated by single-screw extrusion [12]. However, most existing studies focus on producing mushroom-based meat analogues via low-moisture extrusion and are limited to investigating their physicochemical and nutritional properties. To our knowledge, studies integrating comprehensive structural and flavor analysis in Lion’s Mane mushroom-based high-moisture meat analogues are still limited.

Lion’s Mane Mushroom (*Hericium erinaceus*), recognized as a dual-purpose fungus valued for both food and medicinal uses, is prized for its palatable taste, exceptional nutritional richness, and notable antioxidant properties [13]. Additionally, it contains an exceptionally high dietary fiber content. Moderate incorporation of dietary fiber-rich ingredients can improve the rheological properties of raw material systems [14], thereby promoting the development of fibrous structures in meat analogues. Accordingly, this study used soy protein isolate (SPI) as the base material and incorporated Lion’s Mane Mushroom powder (LMM) to produce high-moisture meat analogues (HMMAs) via twin-screw extrusion. Based on this premise, the research investigated the effects of varying LMM incorporation levels (0%, 10%, 20%, 30%, 40%) on the physical properties, structural characteristics, and flavor profile of SPI-based HMMAs. The impact of different LMM additions on the physical and structural properties of HMMAs was evaluated by analyzing the rheological properties of raw materials, extrudate color, texture properties, macroscopic and microscopic structures, particle size distribution, thermodynamic properties, and the secondary and tertiary structures of proteins. Furthermore, Headspace Solid-Phase Microextraction Gas Chromatography–Mass Spectrometry (HS-SPME-GC–MS) was employed to analyze volatile compounds in the extrudates, aiming to explore the influence of LMM addition on their flavor characteristics. This investigation offers valuable insights for informing the development of high-moisture, edible fungus-based meat analogues with superior sensory profiles.

## 2. Materials and Methods

### 2.1. Materials

Soy protein isolate (SPI) was provided by HaGaoKe Soy Food Co., Ltd. (Harbin, Heilongjiang, China). The protein content of SPI was 91.2% as measured by the Kjeldahl method. The crude protein content was calculated from the measured nitrogen content using a conversion factor of 6.25. Lion’s mane mushroom (*Hericium erinaceus*) powder was purchased from North Premium Specialty Trading Company (Jiagedaqi, Heilongjiang, China). Analytical grade chemicals and reagents were obtained from Shangbao Biotechnology Co., Ltd. (Shanghai, China), unless otherwise specified.

### 2.2. Preparation and Extrusion of Meat Analogue

SPI and Lion’s mane mushroom (LMM) powder were blended at different concentrations (0%, 10%, 20%, 30% and 40%, *w*/*w*) to obtain homogeneous mixtures. The mixture was mixed in a high-speed mechanical mixer (HBQD-300, Hongbao Machinery Technology Co., Ltd., Changzhou, China) at 500 rpm for 10 min to ensure that LMMP was evenly distributed throughout the SPI. The prepared mixtures were fed into a experimental twin-screw extruder (CLEXTRAL EV-25, Clestero Co., Ltd., Firminy, France) equipped with a cylindrical cooling die (300 mm, L) at the extruder’s end. The maximum capacity of the extruder is 10 kg/h. The extruder had six temperature-controlled zones: The zone temperature along the extrusion direction were set to 30 °C, 60 °C, 90 °C, 120 °C, 140 °C, and 140 °C, respectively. Screw diameter (D) of the extruder was 25 mm, screw length (L) of the extruder was 600 mm, and the L/D ratio was 24:1. The parameters of the extrusion process were set as follows: the mixture was metered into the system by the solids feeder at a constant rate of 2 kg/h, the DI water was fed by a liquid pump at a constant rate of 3 kg/h, and the screw speed of the extruder was set at 300 rpm. The temperature of the cooling die was controlled at 30 °C by a circulating water cooler. The above extrusion parameters were selected based on preliminary trials and previous studies [15]. Following the achievement of a stable extrusion process, extrudates were collected. The color and texture properties of a subset of fresh samples were characterized immediately. Some samples were stored at 4 °C for DSC and flavor testing. The remaining samples were freeze-dried, and the freeze-dried samples were ground and passed through a 149 μm aperture sieve for testing of other indicators.

### 2.3. Rheological Analysis

SPI and LMM were mixed at different ratios (0%, 10%, 20%, 30% and 40%, *w*/*w*), and the resulting 5 g mixture of raw materials was added to deionized water (20 mL), stirred at 1200 r/min for 3 h, and allowed to stand at 4 °C for 24 h to ensure thorough hydration of the samples [15]. Then, the rheological properties were measured using a Discovery HR-2 rheometer (TA Instrument, New Castle, DE, USA), with slight modifications to the methods of a previous study [15]. A 40 mm diameter parallel plate was selected, and the samples were pre-equilibrated at 25 °C for 5 min before being uniformly spread on the stage, with a gap of 1 mm between the stage and the parallel plate. A constant strain of 1% was applied at a constant temperature of 25 °C, and the apparent viscosity from 1 to 100 s^−1^ was measured. Frequency sweep tests were conducted within an angular frequency range of 0.1–100 rad·s^−1^ to obtain and analyze the variations in storage modulus (G′), loss modulus (G″), and loss tangent (tanδ = G″/G′). In the temperature sweep test, the temperature was first gradually increased from 30 °C to 110 °C at a rate of 10 °C/min and held constant at 110 °C for 1 min, then cooled at a rate of 10 °C/min to 30 °C.

### 2.4. Color Properties Analysis

The color of the extrudate was measured using a handheld colorimeter (CR-400, Konica Minolta, Tokyo, Japan). After turning on the instrument, calibrated using a standard whiteboard, fixed the fresh sample on a flat platform to ensure stability, and selected five smooth positions on the sample to measure brightness (L*), redness (a*), and yellowness (b*). Based on the measured L*, a*, and b* values, the browning index (BI) for each sample was calculated using Equation (1).

(1) $$BI=\frac{100}{0.17}\times \left(\frac{a^{*}+1.75\times L^{*}}{5.645\times L^{*}+a^{*}-3.012\times b^{*}}-0.31\right)$$

### 2.5. Texture Profile Analysis (TPA)

Measurements were conducted using the TPA mode of a texture analyzer (TA-XT Plus model, Stable Micro Systems, Godalming, Surrey, UK), including hardness, elasticity, and chewiness, with slight modifications based on the method of a previous study [16]. Fresh samples were cut into 10 mm × 10 mm × 4 mm (L × W × H) blocks and fixed on the stage. The P/36R probe compressed the samples twice to 75% of their original height, with a pre-test speed of 2.0 mm/s, a test speed of 1.0 mm/s, and a post-test speed of 2.0 mm/s. Measurements were replicated five times for each sample.

### 2.6. Fibrous Degree Analysis

The fibrous degree of the extrudate was measured using a texture analyzer (TA-XT Plus model, Stable Micro Systems, UK), with slight modifications based on the method of a previous study [17], the fresh sample was cut into blocks of 10 mm × 10 mm × 4 mm (L × W × H) and fixed on the stage, and the fibrous degree of the extrudate was measured with an HDP/BS probe with the measurement parameters of 75% pressure distance, 2.0 mm/s pre-test speed, 1.0 mm/s test speed, and 2.0 mm/s post-test speed. The fibrous degree was defined as the ratio of the transverse shear force to the longitudinal shear force along the extrusion direction. Measurements were replicated five times for each sample.

### 2.7. Microstructure Observation

The samples were imaged with a scanning electron microscope (S3400N, Hitachi, Tokyo, Japan) to characterize their microstructure. The collected samples were sectioned into 2 mm thick slices along the extrusion direction. These slices were mounted on conductive adhesive tape, sputter-coated with gold for 1 min, and then imaged under an accelerating voltage of 2.0 kV at 1500× magnification.

### 2.8. Thermal Properties Analysis

The thermal properties of the samples were characterized by Differential Scanning Calorimetry (DSC, Q2000, TA Instruments, New Castle, DE, USA). The experimental procedure closely followed that of Yu et al. [18], with only slight adjustments, experiments were performed under a nitrogen purge of 50 mL/min. Samples weighing 6–8 mg were loaded into hermetically sealed aluminum pans, using an empty pan as a reference. The temperature within the crucible was ramped from 25 °C to 200 °C at a heating rate of 10 °C/min. The peak temperature (T_p_) and enthalpy change (_Δ_H) of the samples were determined based on the recorded DSC curves.

### 2.9. Particle Size Distribution (PSD)

A Mastersizer 3000 particle size analyzer (Malvern Panalytical, Malvern, Worcestershire, UK) was employed for the determination of particle size distribution. The experimental procedure was carried out as described by Wang et al. [19], lyophilized sample powder was dispersed in phosphate-buffered saline (PBS; 0.02 mol/L, pH 7.0) to prepare a 1 mg/mL solution. Measurements were performed at an ambient temperature of 25 °C, with a protein refractive index setting of 1.145, and a measurable size range of 0.01–3500 μm. Measurements were replicated five times for each sample.

### 2.10. Protein Secondary Structure Determination

FTIR spectroscopy (Nicolet iS50, Thermo Fisher Scientific Inc., Waltham, MA, USA) was employed to analyze the protein secondary structure. The freeze-dried sample powder (1 mg) was thoroughly mixed with potassium bromide (KBr; 100 mg) and then pressed into a transparent pellet. Spectra were acquired over the wavenumber range of 4000–400 cm^−1^ with a resolution of 4 cm^−1^, averaging 32 scans per spectrum. Spectral preprocessing and analysis focused on the amide I region (1600–1700 cm^−1^). The spectra within this region were subjected to baseline correction and smoothing using OMNIC 9.2 software. Subsequently, Fourier deconvolution and Gaussian peak fitting were performed using PeakFit 4.12 software to quantify changes in protein secondary structure.

### 2.11. Protein Tertiary Structure Determination

Fluorescence spectroscopy (F-4700, Hitachi High-Tech Corporation, Tokyo, Japan) was utilized to analyze changes in the tertiary structure of the proteins. The experimental procedure closely followed that of Xiao et al. [20], with only slight adjustments, lyophilized samples were first pulverized and sieved. The processed powder was then dissolved in phosphate buffer (0.1 mol/L, pH 7.0) to prepare a 0.3 mg/mL sample solution. The solution was stirred for 10 min and subsequently centrifuged at 3000× *g* for 15 min; the supernatant was collected for analysis. Fluorescence emission spectra were recorded on a spectrofluorometer with an excitation wavelength of 290 nm, scanning the emission range from 300 to 500 nm (slit width = 5 nm for both excitation and emission monochromators). The fluorescence spectrum of the phosphate-buffered solution was used as the blank reference.

### 2.12. HS-SPME-GC–MS Analysis

#### 2.12.1. HS-SPME-GC–MS Conditions

The HMMAs supplemented with 20% LMM (HMMAs-20%LMM), selected as the representative sample, was subjected to volatile flavor component analysis by HS-SPME-GC–MS, while the HMMAs without LMM (HMMAs) served as the control. The methodology was adapted from Song et al. [21] with slight modifications. 500 mg of the sample after liquid nitrogen grinding transferred into the 20 mL headspace bottle, and 10 μL of 2-Octanol were added as internal standard. Following rapid sealing of the headspace vial with a polytetrafluoroethylene septum, the sample was vortexed thoroughly. Volatile compounds were then sampled using a solid-phase microextraction (SPME) fiber (50/30 µm DVB/CAR/PDMS; Supelco, Bellefonte, PA, USA). In the SPME cycle of the PAL rail system, the incubation temperature was set at 60 °C, with a preheating time of 15 min and an incubation time of 30 min, while the desorption time was 4 min. GC-MS analysis was conducted using an Agilent 7890 gas chromatograph coupled to a 5977B mass spectrometer, equipped with a DB-Wax column. The injection was performed in splitless mode. Helium served as the carrier gas, with a front inlet purge flow of 3 mL min^−1^ and a column flow rate of 1 mL min^−1^. The temperature program was as follows: the initial temperature was maintained at 40 °C for 4 min, then increased to 245 °C at a rate of 5 °C min^−1^, and held at this temperature for 5 min. The temperatures of the injection port, transfer line, ion source, and quadrupole were set to 250, 250, 230, and 150 °C, respectively. Electron impact ionization mode was employed with an energy of 70 eV. Mass spectrometry data were acquired in full scan mode with a mass range of *m*/*z* 20–400, and a solvent delay of 2.37 min was applied.

#### 2.12.2. Volatile Components Qualitative and Quantitative Analysis

Chroma TOF 4.3X software of LECO Corporation and Nist database were used for raw peaks exacting, the data baselines filtering and calibration of the baseline, peak alignment, deconvolution analysis, peak identification and peak alignment. Qualitative identification of volatile flavor components was carried out based on mass spectrometry matching, retention time index matching, etc. The retention index of volatile flavor components was calculated by using the retention time of C7-C30 n-alkane mixtures measured under the same conditions as a reference to calculate the retention time of volatile flavor components, thereby obtaining their standard retention index. Relative quantitation of volatile compounds was derived from calibration against an internal standard [22]. An internal standard (o-dichlorobenzene) was used, thereby allowing the relative content of the volatile components to be determined according to Equation (2).

(2) $$W_{i}=f_{i}\times \frac{A_{i}\times m_{s}/A_{s}}{m}$$

W_i_ represents the content of volatile flavor compound i in the sample (µg/kg); f_i_ is the quality correction factor, generally denoted as 1; A_i_ is the peak area of volatile flavor compound i in the sample; A_s_ is the peak area of the internal standard; m_s_ is the mass of the internal standard; m is the mass of the sample.

#### 2.12.3. Analysis of Relative Odor Activity Values

Relative Odor Activity Value (ROAV) was used to quantitatively evaluate the contribution of various volatile flavor compounds to the overall flavor of the test sample, and to determine key volatile flavor compounds [23]. The ROAV value of the volatile flavor compound that contributes most to the overall flavor of the sample set (ROAV_max_) was fixed at 100. The ROAV values of the remaining volatile flavor components, determined by Equation (3).

(3) $${ROAV}_{i}=\frac{C_{i}}{C_{max}}\times \frac{T_{i}}{T_{max}}\times 100$$

ROAV_i_ represents the ROAV value of the volatile flavor compound i in the sample; C_i_ represents the relative percentage content of volatile flavor component i; T_i_ represents the corresponding odor threshold of volatile flavor component i; C_max_ represents the relative percentage content of volatile flavor components that contribute the most to the overall flavor of the sample.

### 2.13. Statistical Analysis

A minimum of three independent replicates were performed for all measurements. Results are expressed as the mean ± standard deviation (SD). Statistical analysis was conducted using SPSS 19.0 software, performing analysis of variance (ANOVA) followed by Duncan’s multiple range test for post hoc comparisons. Homogeneity of variances was verified using Levene’s test, and data met the assumptions for ANOVA. Data analysis and graphical representations were performed using Origin 2019 software.

## 3. Results and Discussion

### 3.1. Rheological Properties Analysis

**Figure 1:**
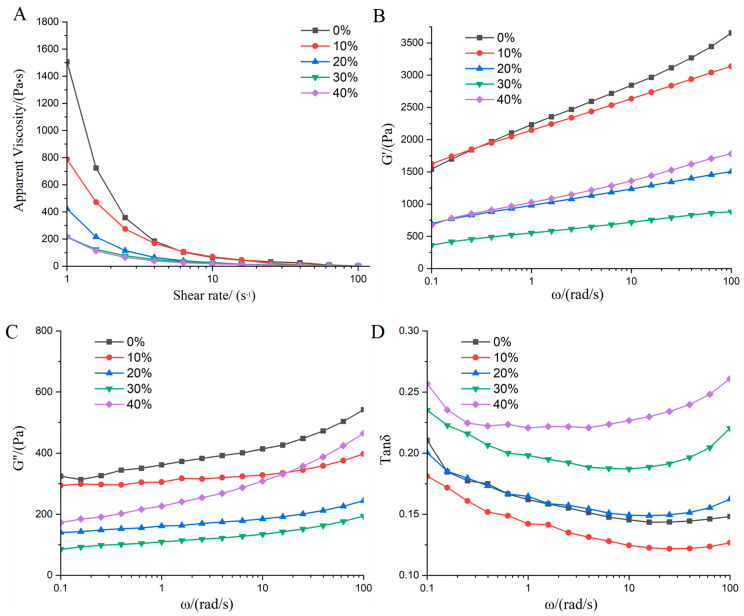
Effects of adding LMM on rheological properties of the dispersions. (**A**) Apparent viscosity. (**B**–**D**) Variation of G′, G″ and tanδ with frequency.

Rheological properties characterize the flow behavior and viscosity of various raw material systems, reflecting the interaction mechanisms between their components [24]. As shown in Figure 1A, all samples exhibited typical shear-thinning behavior, where apparent viscosity decreased with increasing shear rate, indicating non-Newtonian fluid characteristics [25]. The apparent viscosity of the raw material system initially decreased but subsequently increased with the addition of LMM. This initial decrease occurred because LMM contains a large amount of insoluble dietary fiber (IDF), which restricts the water-binding capacity of SPI, thereby reducing system viscosity. However, at 40% LMM incorporation, the apparent viscosity increased. The high β-glucan content in LMM is a key factor contributing to this observed increase; at this level, the elevated β-glucan concentration enhances viscosity due to its strong water-holding capacity and colloid-forming ability. To further explore the impact of LMM on the system’s viscoelasticity, frequency sweep tests were conducted. Results for storage modulus (G′), loss modulus (G″), and loss tangent (tan δ) are presented in Figure 1B–D, respectively. G′ and G″ represent the elastic and viscous components, respectively. Both G′ and G″ values increased with frequency, suggesting the gradual unfolding of protein molecules during oscillation. Furthermore, G′ consistently exceeded G″ across the tested frequency range, indicating viscoelastic gel behavior with dominant elastic properties [26]. The addition of LMM caused G′ and G″ values to first decrease and then increase, consistent with the apparent viscosity trend. This behavior is primarily due to the opposing effects of IDF and β-glucan present in LMM. At lower addition levels (≤20%), the insoluble dietary fiber (IDF) acts as a disruptive filler, interrupting the continuous soy protein network and weakening the gel structure, thereby reducing the moduli. However, at a high addition level (40%), the high concentration of water-soluble β-glucan dominates the system’s rheology. Its strong water-binding and gel-forming capabilities allow it to establish a percolating network, which reinforces the overall matrix and leads to a recovery and eventual increase in both G′ and G″. Throughout the experiment, the tan δ values did not exceed 1, as shown in Figure 1D, confirming that G′ > G″ (elasticity > viscosity) throughout. This characteristic is beneficial for forming a strong gel structure during subsequent extrusion. When the addition amount of LMM is 0%, the value of G′ is 2339.55 Pa, the value of G″ is 321.23 Pa, and the value of tanδ is 0.14, while when the addition amount of MM increases to 40%, the value of G′ is 1173.24 Pa, the value of G″ is 270.13 Pa, and the value of tanδ is 0.22. The increase in tanδ indicates the change from elastic to viscous behavior. These values quantify changes in rheological properties and are consistent with the trends described in the text. These findings demonstrate that incorporating LMM alters the rheological behavior of the initial formulation, consequently influencing the physicochemical properties of the extrudates.

### 3.2. Effects of LMM Content on the Apparent Properties

#### 3.2.1. Texture Properties Analysis

The texture of meat analogues is a critical factor influencing consumer acceptance and is typically evaluated through textural properties including hardness, springiness, chewiness, and fibrous degree [27]. The fibrous degree is an index that characterizes the attainment of a meat-like fibrous texture in the extrudates, defined as the ratio of longitudinal shear force (perpendicular to the extrusion direction) to transverse shear force (parallel to the extrusion direction) [16]. This dimensionless value is greater than 1 [28], and an increase directly reflects enhanced fibrous structure formation. As shown in Table 1, incorporating LMM into SPI increased the fibrous degree of the extrudates. As the LMM content increased from 0 to 40%, the fibrous degree exhibited an initial increase followed by a subsequent decrease. Extrudates produced using SPI alone exhibited the lowest fibrous degree (1.05), indicating a poor fibrous structure. At 20% LMM incorporation, the fibrous degree reached its peak value of 1.54, demonstrating that an appropriate amount of LMM promotes fibrous structure formation. Relevant studies indicate that two or more incompatible phases can inhibit lateral protein aggregation, favoring longitudinal alignment and fibrous structure formation [29]. In this study, LMM acted as a second phase introduced into the SPI matrix, creating two incompatible and distinct phases. Consequently, at optimal LMM levels, it prevented lateral protein aggregation and promoted longitudinal alignment. However, when LMM addition reached 30% or more, the fibrous degree decreased, indicating that excessive LMM hindered fibrous structure formation. This is likely attributed to the high insoluble dietary fiber (IDF) content in LMM. Excessive LMM addition significantly increases IDF levels, which disrupts the continuity of the protein matrix, resulting in a fractured and discontinuous fibrous network. The fibers become short, coarse, and disordered [30], thereby adversely affecting the fibrous structural quality of the extrudates.

Texture profile analysis (TPA) measurements were conducted on the extrudates using a texture analyzer to assess hardness, springiness, and chewiness. As shown in Table 1, incorporating LMM (0–40%) into SPI resulted in a decreasing trend in extrudate hardness and chewiness, while springiness showed no significant changes. At 0% LMM addition, the extrudates exhibited the highest hardness and chewiness values (7044.55 g and 5394.12 g, respectively). As the LMM content increased, both hardness and chewiness gradually decreased. These results indicate that adding LMM to SPI produces extrudates with a softer texture. This is likely because SPI tends to form hard and brittle gels [31], whereas LMM contains a high amount of insoluble dietary fiber (IDF). The IDF disrupts the original SPI gel network, consequently leading to textural softening and a concomitant reduction in hardness and chewiness. Overall, the findings demonstrate that an appropriate amount of LMM (20%) can enhance the textural attributes of the extrudates.

#### 3.2.2. Color Analysis

Color is a critical sensory attribute of meat analogues, primarily influenced by the Maillard reaction during extrusion and the inherent color of the raw materials [32]. To assess the impact of Lion’s Mane Mushroom (LMM) powder addition on the color properties of extrudates, their color parameters (L*, a*, b*, and BI) were analyzed. According to the results presented in Table 1, extrudates produced using SPI alone exhibited the lowest BI value (48.77). However, the BI increased with higher LMM content, indicating that LMM incorporation resulted in darker extrudates. Simultaneously, both a* (redness/greenness) and b* (yellowness/blueness) values increased with increasing LMM levels, signifying a shift toward a more reddish-yellow hue. This color shift can be attributed to two main factors:

Inherent Color: LMM has a yellowish-brown hue that is darker than SPI, which directly affects the final color of the extrudate.

Enhanced Maillard Reaction: The Maillard reaction, a key factor influencing extrudate color during high-moisture extrusion, involves interactions between free amino groups and reducing sugars under high-temperature conditions [27]. LMM, which is rich in polysaccharides, promotes the Maillard reaction during extrusion, resulting in intensified browning and a darker extrudate color.

### 3.3. Microstructure and Visual Observation

**Figure 2:**
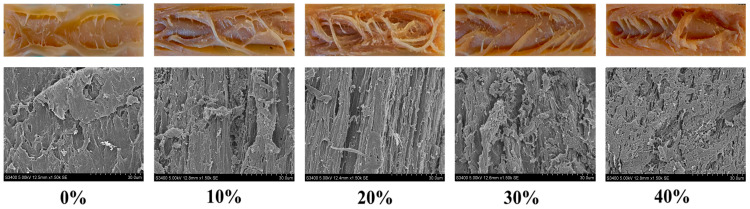
SEM and visual images of SPI-LMM extrudates.

Scanning electron microscopy (SEM) serves as a powerful tool for characterizing the microstructural features of plant-based meat analogue products [33]. In this study, extrudates with varying levels of LMM incorporation were analyzed using SEM. Combined with photographic documentation of the extrudates’ fibrous structures, these observations enabled a comparison of the effects of different LMM addition levels on the microstructure of the plant-based meat analogues (Figure 2). Macroscopic examination of the samples revealed that extrudates produced with SPI alone failed to form distinct fibrous structures. Corresponding microstructural observations confirmed that SPI-only extrudates exhibited a relatively dense gel structure. However, the incorporation of LMM resulted in the emergence of observable fibrous structures in the extrudates. Extrudates containing 20% LMM displayed the most pronounced and well-defined fibrous structure. As the LMM content increased beyond this level, the fibrous structure became less distinct and showed an increased presence of lamellar structures. This observation aligns with earlier measurements of fibrous degree. These findings demonstrate that an optimal amount of LMM promotes the formation of fibrous structures in the extrudates. The primary mechanism involves LMM acting as a second phase within the SPI matrix during extrusion, creating two incompatible and distinct phases. These incompatible phases inhibit lateral protein aggregation, thereby favoring longitudinal alignment and fiber formation [34]. Therefore, adding an appropriate amount of LMM to SPI facilitates fibrous structure development. Conversely, excessive LMM addition impedes fiber formation, resulting in a looser structure. This detrimental effect is likely attributable to the high insoluble dietary fiber (IDF) content in LMM. The surplus IDF disrupts the continuity of the protein network [30], which impeded the development of well-aligned fibrous structures along the extrusion axis. At the same time, the observation of the microstructure of the extrudates through SEM (Figure 1) provides insight into its texture properties. When the LMM addition was 0%, the image showed excellent compaction and adhesion between the fiber and the polymer matrix, with no visible voids. This continuous and well-compacted structure results in a high hardness and chewiness of the extrudates. When the LMM addition was 20%, the fibers exhibited a high degree of arrangement in the extrusion direction, which facilitated efficient stress transfer. Microscale pores are visible within the matrix as LMM increases, and pores are more pronounced when LMM is added greater than 20%, and these structures can lead to a decrease in extrudates hardness and chewiness. This is consistent with the results of the texture properties of the extrudates mentioned earlier.

### 3.4. Thermal Behavior Analysis

**Figure 3:**
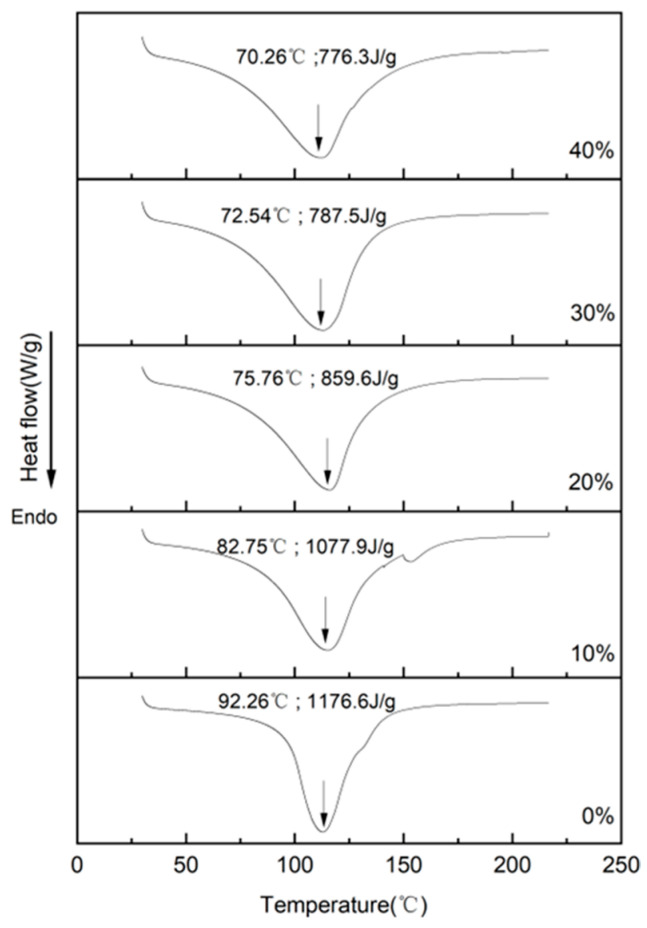
DSC thermograms of SPI-LMM extrudates.

The formation of fibrous structures during high-moisture extrusion (HME) is primarily driven by protein denaturation, a process governed by specific energy inputs [16]. Differential Scanning Calorimetry (DSC) evaluates protein behavior during HME by monitoring the peak temperature (T_p_) and enthalpy change (_Δ_H), which serve as indicators of conformational thermal stability and the extent of aggregation [35]. Figure 3 presents the DSC curves, T_p_, and _Δ_H values for SPI-LMM extrudates with varying levels of LMM incorporation. All curves exhibited a single major endothermic peak. Furthermore, as shown in Figure 3, both the peak temperature (T_p_) and denaturation enthalpy (_Δ_H) of all samples decreased with increasing LMM content. Extrudate raw material systems containing only SPI exhibited the highest T_p_ and _Δ_H values (92.26 °C and 1176.6 J/g, respectively). Conversely, when LMM addition reached 40%, both T_p_ and _Δ_H reached their lowest values (70.26 °C and 776.3 J/g, respectively). This phenomenon is attributed to the insoluble dietary fiber (IDF) present in LMM. Higher levels of IDF hinder the formation of the protein network structure during the cooling and shaping phases [30]. This disruption consequently reduces the thermal stability of the extrudates, leading to the observed decreases in T_p_ and _Δ_H.

### 3.5. Particle Size Analysis

**Figure 4:**
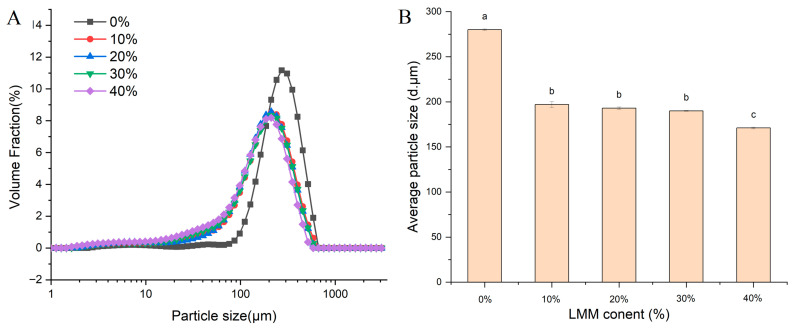
(**A**) Particle size distribution of SPI-LMM extrudates. (**B**) Average particle size of SPI-LMM extrudates. Note: Different letters of the same indicator mean significant differences (*p* < 0.05).

Particle size is a crucial indicator for evaluating protein aggregation behavior during HME. As illustrated in Figure 4, the incorporation of LMM exerted a significant influence on the particle size distribution (PSD) of the extrudates. All samples exhibited a unimodal distribution, predominantly within the 10–1000 μm range. Increasing the LMM content in the SPI blend shifted the PSD toward smaller particle sizes, indicating a gradual reduction in the mean particle size. This phenomenon likely results from the combined effects of two primary mechanisms:

Microphase Separation: LMM contains a substantial amount of insoluble dietary fiber (IDF). The poor compatibility between LMM and SPI promotes microphase separation, resulting in two distinct, incompatible phases [29]. This phase separation inhibits the formation of large protein aggregates, thereby reducing the average particle size.

Steric and Hydrophobic Effects: LMM is rich in β-glucan. As its concentration increases, the elevated β-glucan levels create greater steric hindrance between protein molecules. Concurrently, this hinders the exposure and aggregation of hydrophobic groups [20]. Both effects impede the formation of larger protein aggregates, contributing to the observed decrease in mean particle size.

As shown in Figure 4B, while the mean particle sizes for samples containing 10% to 40% LMM appear visually close, they represent a new, stable state of the system significantly different from the 0% control. Beyond the initial 10% threshold, the particle size reduction reached a plateau. This suggests a dynamic equilibrium was established: the insoluble dietary fiber (IDF) continued to drive phase separation, while the released β-glucans exerted a counteracting steric hindrance effect, preventing the particles from coalescing and thus stabilizing the particle size within a specific range.

### 3.6. Protein Secondary Structure Analysis

**Figure 5:**
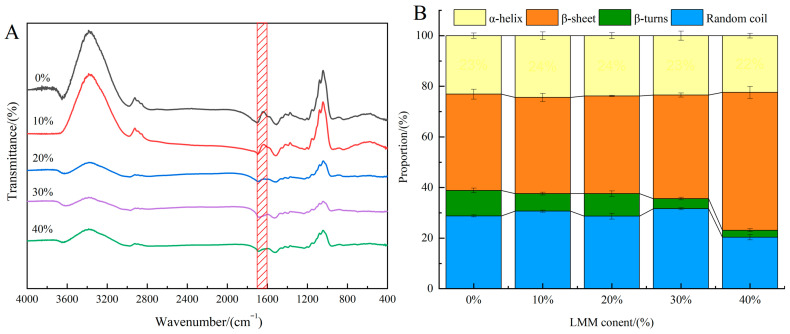
(**A**) FTIR curves for SPI-LMM extrudates; (**B**) secondary structure percentage of SPI-LMM extrudates. Note: Different letters of the same indicator mean significant differences (*p* < 0.05).

Fourier Transform Infrared (FTIR) spectroscopy serves as a valuable tool for characterizing changes in protein secondary structure [20]. A comparison of the FTIR spectra for extrudates with varying LMM incorporation levels is presented in Figure 5A. The amide I band (1600–1700 cm^−1^) is the most informative region of the FTIR spectrum, where specific wavenumber ranges correspond to distinct protein secondary structures: α-helix (1646–1664 cm^−1^), β-sheet (1615–1637 cm^−1^ and 1682–1700 cm^−1^), β-turn (1664–1681 cm^−1^), and random coil (1637–1645 cm^−1^) [16]. As shown in Figure 5B, the quantitative distribution of these secondary structures was determined by Gaussian curve fitting and second-derivative analysis of this band employing PeakFit 4.12 software, revealing the impact of LMM addition on the extrudates’ secondary structure. β-sheets and α-helices represent tightly ordered structures stabilized by intramolecular hydrogen bonds, whereas β-turns and random coils are loose, disordered conformations [36]. All extrudates exhibited these four secondary structure elements (Figure 5B), predominantly comprising ordered structures (α-helix + β-sheet > 60%). The β-sheet content significantly exceeded the α-helix content, indicating that SPI-based extrudates primarily adopt ordered secondary structures, consistent with prior research [36]. Notably, the relative content of β-sheets showed a significant increasing trend with higher LMM levels. Since β-sheets maintain their conformation via intramolecular hydrogen bonds, this increase suggests enhanced intramolecular hydrogen bonding within the extrudates. This phenomenon is likely attributable to the substantial β-glucan content in LMM. During extrusion, increased β-glucan levels strengthen intramolecular hydrogen bonding forces within SPI [20]. Consequently, it can be inferred that LMM incorporation facilitates the conformational rearrangement of SPI molecules during high-moisture extrusion, promoting a transition from disordered structures (β-turn and random coil) toward ordered structures (α-helix and β-sheet).

### 3.7. Protein Tertiary Structure Analysis

**Figure 6:**
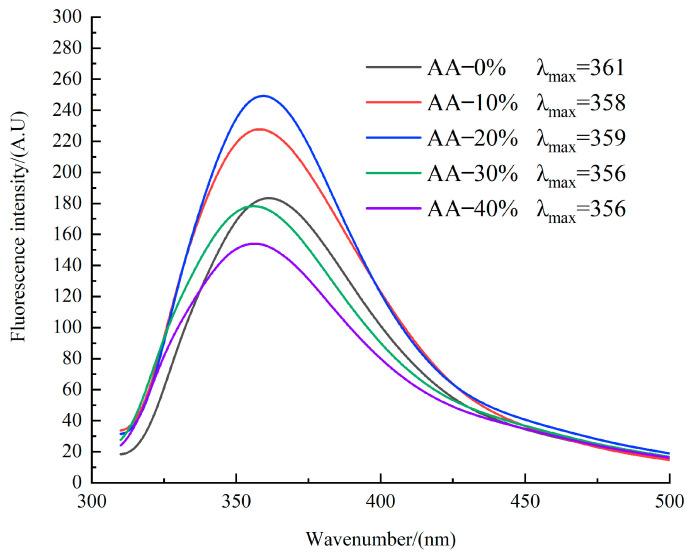
Fluorescence spectra curves for SPI-LMM extrudates.

Tryptophan (Trp) residues serve as intrinsic fluorescent probes that are highly sensitive to alterations in their local microenvironment, reflecting protein conformational changes. Subtle alterations in their microenvironment can be detected through variations in fluorescence intensity and shifts in the maximum emission wavelength (λ_max_) [37]. Consequently, intrinsic fluorescence spectroscopy effectively reflects conformational changes in the tertiary structure of proteins [35]. A λ_max_ value below 330 nm indicates that Trp residues are located in a hydrophobic environment, whereas a λ_max_ value above 330 nm signifies a hydrophilic environment [38]. As shown in Figure 6, the tryptophan residues in SPI samples were situated in hydrophilic environments across all treatments. The fluorescence intensity initially increased and then decreased with increasing LMM incorporation, accompanied by a blue shift in λ_max_.

At LMM levels ≤ 20%, fluorescence intensity gradually increased with the addition of LMM. This finding is consistent with the conformational unfolding of protein molecules, leading to the gradual exposure of previously buried tryptophan side chains [39]. This phenomenon is attributed to the high insoluble dietary fiber (IDF) content in LMM. The incorporation of IDF disrupts the continuity of the protein matrix, causing fractures and discontinuities within the fibrous network, thereby exposing more tryptophan residue side chains.

At LMM levels above 20%, fluorescence intensity progressively decreased with further LMM addition. This reduction indicates that excessive LMM diminishes the exposure of tryptophan residues and masks the chromophores, resulting in decreased fluorescence intensity—a phenomenon known as static quenching [19]. This effect likely arises from two mechanisms related to the high β-glucan content in LMM:

Covalent Interactions: Under the high-temperature, high-pressure, and high-shear conditions of extrusion, β-glucan can form irreversible covalent bonds with proteins, significantly reducing the accessible tryptophan content.

Steric Hindrance: β-glucan exerts a steric shielding effect by blocking the fluorescence signal from the tryptophan residues in soy protein, thereby reducing fluorescence intensity [20].

At LMM addition ≤ 20%, the exposure of tryptophan residues (increased fluorescence intensity and blue shift) implies the unfolding of protein molecules and the unfolding of tertiary structures. This structural unfolding often favors proteases such as pepsin and trypsin to approach their cleavage sites, potentially increasing the initial rate of protein digestion. Conversely, when the LMM addition > 20%, fluorescence quenching (static quenching) indicates that the protein forms a complex with the β-glucan in the LMM, which may physically impede the protease’s contact with the protein substrate. We will infer from this that excessive addition of LMM may lead to a decrease in protein digestibility.

### 3.8. Volatile Flavor Components Analysis

**Figure 7:**
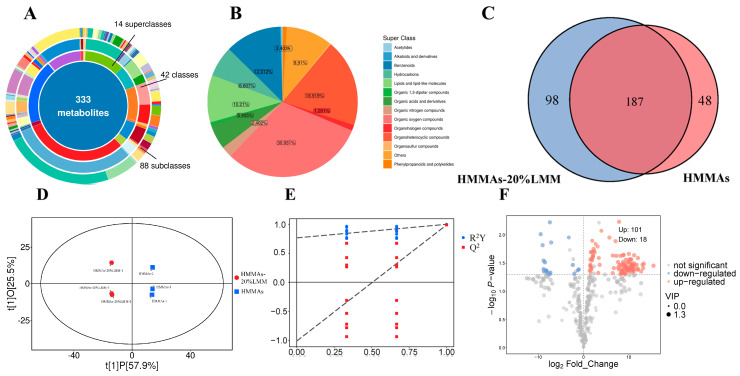
Comprehensive analysis of volatile flavor components: (**A**) classification of volatile flavor components; (**B**) proportion of volatile flavor components; (**C**) types of volatile flavor metabolites Venn diagram; (**D**) score scatter plot for PCA model TOTAL; (**E**) permutation plot test of OPLS-DA model; (**F**) volcano plot for differentially expressed metabolites.

Analysis by HS-SPME-GC-MS revealed the presence of 333 volatile flavor compounds, categorized into 14 distinct classes, in both HMMAs-20%LMM and HMMAs (Figure 7A). These classes included Acetylides (1 compound), Alkaloids and derivatives (1 compound), Benzenoids (40 compounds), Hydrocarbons (22 compounds), Lipids and lipid-like molecules (34 compounds), Organic 1,3-dipolar compounds (2 compounds), Organic acids and derivatives (18 compounds), Organic nitrogen compounds (8 compounds), Organic oxygen compounds (103 compounds), Organohalogen compounds (4 compounds), Organoheterocyclic compounds (63 compounds), Organosulfur compounds (1 compound), Phenylpropanoids and polyketides (3 compounds), and Other compounds (33 compounds) (Figure 7B). Specifically, HMMAs-20%LMM contained 285 volatile flavor compounds, while HMMAs contained 235. Both shared 187 common compounds. Compared to HMMAs, HMMAs-20%LMM exhibited 98 newly identified volatile flavor compounds and a loss of 48 compounds (Figure 7C).

Orthogonal Projections to Latent Structures Discriminant Analysis (OPLS-DA) was conducted on the detected volatile flavor compounds (Figure 7D). The results demonstrated a model fit with R^2^X (explained variance in X) of 0.835, R^2^Y (explained variance in Y) of 1, and a predictive ability Q^2^ of 0.989. The two key model parameters, R^2^Y and Q^2^ values exceeding 0.5 indicate excellent separation between the sample groups [40]. A permutation test (200 iterations, Figure 7E) confirmed the model’s validity, as the Q^2^ regression line intercept on the *Y*-axis was below zero, indicating no overfitting. These results clearly indicate a significant difference in the overall flavor profile between HMMAs-20%LMM and HMMAs (Control). To identify characteristic flavor compounds distinguishing HMMAs-20%LMM from HMMAs, differential metabolites were identified according to the following criteria: Variable Importance in Projection (VIP) > 1, *p*-value < 0.05, and Fold Change (FC) ≥ 2 or FC ≤ 0.5. This screening identified 119 differential metabolites. Compared to HMMAs, HMMAs-20%LMM exhibited upregulation of 101 volatile flavor compounds. The majority belonged to the organic oxygen compounds class (28 compounds), primarily aldehydes, ketones, and alcohols. Aldehydes typically impart grassy, fatty, and almond notes [41]; ketones contribute fruity and creamy aromas [42]; and alcohols enhance mushroom-like and grassy characteristics [43]. Conversely, 18 compounds were downregulated (Figure 7F). Thus, HMMAs-20%LMM possesses a significantly more abundant and complex volatile flavor profile compared to the control HMMAs. 

### 3.9. Relative Odor Activity Values (ROAV) Analysis

In all tested samples, the Relative Odor Activity Value (ROAV) for each compound was ≤100, with higher ROAV values indicating a greater contribution to the overall flavor profile. Compounds with ROAV ≥1 are defined as key aroma compounds, while those with 0.1 ≤ ROAV <1 function as flavor modifiers. Since 4-mercapto-4-methyl-2-pentanone exhibited the highest peak intensity among all volatile flavor compounds and contributed most significantly to the overall odor, its ROAV was normalized to 100 for calculating the ROAVs of other volatiles (key compounds are listed in Table 2). Analysis revealed that HMMAs-20%LMM and HMMAs contained 7 and 6 key volatile flavor compounds, respectively, sharing five common compounds: 4-mercapto-4-methyl-2-pentanone (black currant flavor), hexanal (fresh apple flavor), 1-octen-3-ol (mushroom flavor), heptanal (fatty flavor), and nonanal (oily flavor). Compared to HMMAs, HMMAs-20%LMM gained two new key volatiles—benzaldehyde (bitter almond and caramel flavor) and 2-heptanone (blue cheese and nutty flavor)—but lost 2-methylbutyraldehyde (almond and malt flavor). The top three components with the highest ROAV values for both HMMAs and HMMAs-20%LMM were 4-mercapto-4-methyl-2-pentanone, hexanal, and 1-octen-3-ol. 4-Mercapto-4-methyl-2-pentanone, which often imparts a black currant aroma with a slight meaty note [44], and hexanal, known for its grassy scent and reported to contribute a greasy, meaty character similar to other aldehydes [45], collectively contributed to the meat-like aroma. Additionally, 1-octen-3-ol, characterized by a mushroom-like flavor and identified as a key aroma compound in numerous edible mushrooms [46], imparted a distinct fungal note to both HMMAs and HMMAs-20% LMM. The cumulative ROAV of key compounds in HMMAs-20%LMM substantially exceeded that of HMMAs, driven primarily by the remarkably high ROAV (18.043) of 1-octen-3-ol. Consequently, HMMAs-20%LMM exhibits enhanced flavor complexity and a more pronounced mushroom character relative to HMMAs.

## 4. Conclusions

This study investigated the impact of LMM as a functional additive on the quality of high-moisture meat analogues (HMMAs) based on soy protein isolate (SPI), prepared via twin-screw extrusion with varying LMM incorporation levels (0%, 10%, 20%, 30%, and 40%). Comprehensive analyses were conducted on the rheological properties of raw materials, product texture, microstructure, thermal stability, protein conformation, and flavor profiles. The addition of LMM significantly altered rheological behavior: at lower concentrations (≤20%), insoluble dietary fiber (IDF) reduced system viscosity, whereas at higher levels (40%), the water-holding capacity and colloid-forming ability of β-glucans increased viscosity. Texture analysis revealed a significant decrease in hardness and chewiness with increasing LMM, attributed to IDF weakening the SPI gel network and imparting a softer texture. The fibrous degree peaked at 20% LMM (1.54), as LMM acted as a second phase to inhibit protein lateral aggregation and promote longitudinal fiber alignment; however, excessive LMM (≥30%) disrupted protein network continuity via IDF, causing structural fragmentation. Scanning electron microscopy (SEM) confirmed optimal fibrous structure formation at 20% LMM, while higher levels induced lamellar structures and disorganized arrangements. The observed decreases in denaturation temperature and enthalpy via DSC suggest that IDF interferes with the development of the protein network upon cooling. Particle size distribution analysis demonstrated that LMM-induced microphase separation and β-glucan steric hindrance decreased average particle size. Fourier-transform infrared spectroscopy (FTIR) revealed that LMM promoted an increase in β-sheet content, facilitating a transition from disordered structures (β-turns, random coils) to ordered conformations. Fluorescence spectroscopy showed that lower LMM levels (≤20%) exposed tryptophan residues (increased fluorescence intensity), while higher concentrations (>20%) caused static quenching (decreased intensity) via β-glucan covalent binding and steric shielding. Gas chromatography-mass spectrometry (GC-MS) equipped with a headspace solid-phase microextraction (HS-SPME) system identified 285 volatile compounds (versus 235 in the control), including 98 newly generated species (primarily aldehydes, ketones, and alcohols). Key flavor compounds in HMMAs with 20% LMM—1-octen-3-ol (mushroom-like, ROAV = 18.043), benzaldehyde (bitter almond), and 2-heptanone (nutty)—imparted a pronounced mushroom flavor. Orthogonal partial least squares-discriminant analysis (OPLS-DA) confirmed significant flavor profile distinctions from the control (R^2^Y = 1, Q^2^ = 0.989). The study established 20% LMM as the optimal incorporation level, synergistically enhancing fibrous degree and textural palatability while reducing hardness. LMM enriched volatile compound profiles and provided natural masking of undesirable plant protein flavors via mushroom-characteristic compounds (e.g., 1-octen-3-ol). These results offer a foundation for optimizing the sensory attributes (texture and flavor) and nutritional profile of SPI-based HMMAs, offering theoretical insights for fiber structure design in high-moisture extrusion and advancing the development of sustainable, health-oriented fungal–plant protein composite meat analogues. However, this research primarily focuses on the physicochemical and structural properties of HMMA. To further validate the practical applicability of LMM-reinforced meat analogues, future studies will conduct comprehensive nutritional analyses on meat analogues supplemented with LMM, including amino acid score and vitamin and mineral composition, to thoroughly evaluate the nutritional value and potential health benefits of the final product. Furthermore, formal sensory evaluation studies conducted by trained team members are essential for objectively quantifying sensory characteristics (such as texture, flavor, aroma, and overall acceptability). These studies are crucial for bridging the gap between laboratory-scale production and commercial adoption.

## Figures and Tables

**Table 1:** The effect of LMM content on the texture and color properties of SPI-LMM extrudates.

LMM Content (%)	Fibrous Degree	Hardness (g)	Springiness	Chewiness (g)	L*	A*	B*	Browning Index (BI)
0	1.05 ± 0.03 ^d^	7044.55 ± 144.10 ^a^	0.96 ± 0.02 ^a^	5394.12 ± 298.25 ^a^	45.54 ± 0.61 ^a^	3.46 ± 0.20 ^c^	16.23 ± 0.30 ^d^	48.77 ± 1.43 ^d^
10	1.28 ± 0.01 ^b^	6501.09 ± 314.92 ^b^	0.94 ± 0.01 ^a^	4750.47 ± 211.31 ^b^	45.05 ± 0.33 ^ab^	4.94 ± 0.26 ^b^	18.75 ± 0.60 ^c^	61.75 ± 1.25 ^c^
20	1.54 ± 0.01 ^a^	5574.34 ± 506.08 ^c^	0.95 ± 0.03 ^a^	4092.60 ± 294.57 ^c^	44.52 ± 0.68 ^b^	7.15 ± 0.53 ^a^	22.09 ± 0.62 ^b^	80.83 ± 0.42 ^b^
30	1.26 ± 0.05 ^b^	4691.55 ± 383.83 ^d^	0.92 ± 0.04 ^a^	3192.07 ± 242.24 ^d^	45.61 ± 0.08 ^a^	7.73 ± 0.22 ^a^	23.18 ± 0.16 ^a^	81.20 ± 0.50 ^b^
40	1.19 ± 0.02 ^c^	3665.08 ± 417.57 ^e^	0.94 ± 0.06 ^a^	2536.08 ± 395.64 ^e^	45.06 ± 0.48 ^ab^	7.76 ± 0.23 ^a^	23.06 ± 0.20 ^a^	82.07 ± 1.66 ^a^

Note: Different letters within the same group mean significant difference (*p* < 0.05).

**Table 2:** ROAV of the key volatile flavor components.

No.	Compound	Threshold (μg/g)	Odor Description	ROVA	
				HMMAs-20%LMM	HMMAs
1	4-mercapto-4-methyl-2-pentanone	0.000005	black currant flavor	100.000 **	100.000 **
2	Hexanal	0.0075	grass flavor	30.748 **	7.779 **
3	1-Octen-3-ol	0.007	mushroom flavor	18.043 **	2.874 **
4	Benz aldehyde	0.00075	bitter almond flavor and caramel flavor	4.568 **	0.725 *
5	2-Heptanone	0.2	blue Cheese flavor and nuts flavor	4.456 **	0.756 *
6	Heptanal	0.01	fatty flavor	1.967 **	1.034 **
7	Nonanal	0.015	oily flavor	1.910 **	2.521 **
8	2-Methylbutyraldehyde	0.003	almond flavor and malt flavor	0.367 *	1.105 **

Note: ** key flavor compounds (ROAV ≥ 1); * flavor compounds with modifying effects (0.1 ≤ ROAV < 1).

## Data Availability

The original contributions presented in this study are included in the article. Further inquiries can be directed to the corresponding author.
